# Graph-Powered Interpretable Machine Learning Models for Abnormality Detection in Ego-Things Network

**DOI:** 10.3390/s22062260

**Published:** 2022-03-15

**Authors:** Divya Thekke Kanapram, Lucio Marcenaro, David Martin Gomez, Carlo Regazzoni

**Affiliations:** 1 Department of Electrical, Electronics and Telecommunication Engineering and Naval Architecture, University of Genova, 16145 Genova, Italy; lucio.marcenaro@unige.it (L.M.); carlo.regazzoni@unige.it (C.R.); 2 Centre for Intelligent Sensing, School of Electronic Engineering and Computer Science (EECS), Queen Mary University of London, London E1 4NS, UK; 3 Intelligent Systems Laboratory, Universidad Carlos III de Madrid, 28903 Madrid, Spain; dmgomez@ing.uc3m.es

**Keywords:** self-awareness, collective-awareness, interpretability, Markov jump particle filter, dynamic Bayesian network, abnormality detection

## Abstract

In recent days, it is becoming essential to ensure that the outcomes of signal processing methods based on machine learning (ML) data-driven models can provide interpretable predictions. The interpretability of ML models can be defined as the capability to understand the reasons that contributed to generating a given outcome in a complex autonomous or semi-autonomous system. The necessity of interpretability is often related to the evaluation of performances in complex systems and the acceptance of agents’ automatization processes where critical high-risk decisions have to be taken. This paper concentrates on one of the core functionality of such systems, i.e., abnormality detection, and on choosing a model representation modality based on a data-driven machine learning (ML) technique such that the outcomes become interpretable. The interpretability in this work is achieved through graph matching of semantic level vocabulary generated from the data and their relationships. The proposed approach assumes that the data-driven models to be chosen should support emergent self-awareness (SA) of the agents at multiple abstraction levels. It is demonstrated that the capability of incrementally updating learned representation models based on progressive experiences of the agent is shown to be strictly related to interpretability capability. As a case study, abnormality detection is analyzed as a primary feature of the collective awareness (CA) of a network of vehicles performing cooperative behaviors. Each vehicle is considered an example of an Internet of Things (IoT) node, therefore providing results that can be generalized to an IoT framework where agents have different sensors, actuators, and tasks to be accomplished. The capability of a model to allow evaluation of abnormalities at different levels of abstraction in the learned models is addressed as a key aspect for interpretability.

## 1. Introduction

Machine learning (ML) theories and applications experienced tremendous growth in the past few decades. Nowadays, ML is extensively used for making accurate predictions in several application areas such as medicine [1], stock markets [2], criminal justice systems [3], telecommunication networks [4], and many others. However, unclear reasoning mechanisms behind an automatic decision often represent one of ML approaches’ drawbacks: this feature is typically referred to as interpretability or explainability of the model. The Internet of Things (IoT) field is expanding faster than its supporting technologies. In IoT applications, the transparency of the model is essential for better interpretability and reliability. The black-box nature of most ML models hinders their applications in important decision-making domains like autonomous vehicles, the financial stock market, or e-health. Therefore, improving ML models’ interpretability is essential to maximize the acceptance of such approaches in these emerging, fast-growing, and promising application fields. This work represents the data-driven ML models using a Bayesian network (BN). A BN is a generative model, i.e., a probabilistic model with a chain of conditional probabilities defined by a group of variables and their links. The links define causal stochastic relationships among BN variables, and the knowledge of such variables allows the model to infer possible states in a probabilistic way. Moreover, a dynamic Bayesian network (DBN) includes the links between the BN variables in different time instants to describe temporal relationships. The proposed approach uses DBNs to model the so-called agents’ self-awareness. Self-awareness (SA) can be defined as a capability of the autonomous system to describe the acquired knowledge about itself and its surroundings and to learn new models incrementally when it experiences new situations [5]. A series of coupled DBNs can represent the interactions between involved agents, thus leading to a possible extension of the model for collective awareness (CA). One of the core functionalities of the self-awareness layer is the capability of incremental learning of new models while the agents experience new situations. The main contributions of the paper can be summarized as follows:

- A novel method is proposed to learn data-driven ML models representing self-awareness and interactive collective awareness of agents’ networks. The learned model is interpretable, i.e., the model is self-explainable about the results it produces and the decisions taken in different situations. For the inferences, a Markov jump particle filter (MJPF) based on generative DBN models is used and extended to become able to detect local and global abnormalities.
- The system incrementally learns new models when the agents encounter new experiences, i.e., when the model detects abnormal situations. The abnormalities generated at different abstraction levels of the models are presented and compared. In this work, interpretability is related to using anomaly data to modify the existing model used to detect the anomaly itself.

The remainder of this paper is structured as follows. The main state-of-the-art contributions regarding intelligent entities and networks are reported and summarized in Section 2. Section 3 explains the proposed strategy for developing a model to detect local and global anomalies by using self-awareness and collective-awareness functionalities. The experimental setup and the datasets are described in Section 4. Section 5 discusses the results obtained at different abstraction levels of the models and the interpretability feature. Conclusions and future work are drawn in Section 6.

## 2. State of the Art

Interpretable machine learning is a fast-growing field and there have been many works done in this research area to examine various aspects of interpretability. In addition to what a model predicts, the ability to interpret the models’ learned knowledge gets a tremendous amount of attention. In [6], the interpretable ML is defined as the ability of ML models to extract knowledge of domain relationships in the data. Moreover, the paper discusses the crucial issues in designing such a model by considering different application areas. On the other hand, the work in [7] provides a survey on the existing state-of-the-art techniques for enhancing the models’ interpretability. According to the definition in [8], interpretability is the degree to which a human can understand the reasoning behind a decision taken by the model. In [9], the author focuses on ML models for relational data and discusses the interpretability of the models’ decisions. This work tackles the challenge of developing intelligence and awareness in agents and then considers the interpretability of artificial intelligence. The interpretability feature takes the ML models to the next level, and it has several consequences in the field of the Internet of Things. The interpretable ML models representing the interactions between agents have many applications in healthcare [10], vehicular networks [11], etc. The works in [12,13] propose the methodology to develop intelligence in agents with the help of ML algorithms and signal processing techniques. The authors used low-dimensional data sequences to build models to detect abnormal situations. Moreover, the work in [14,15] showed a methodology to develop models to represent self-awareness in agents by considering low dimensional as well as high dimensional sensory data. The initial level of interpretability for the ML models represents collective awareness in agents presented in [16]. Most of the existing state-of-the-art approaches lack in model’s interpretability to show which features are used to make automated decisions. Moreover, many of them are focused only on defining the interpretability feature and the critical implementation issues rather than developing interpretable ML models [6,7]. In this work, we propose a methodology to learn a data-driven ML model with self-awareness and collective awareness capabilities to detect local and global anomalies. The interpretability functionality is discussed by giving attention to the results and the model’s decisions as part of emergent incremental learning.

## 3. Interpretable Machine Learning Models: Design and Implementation

This section describes the steps involved in interpretable ML models’ training and testing phases by considering a simple two-ego-things network. Ego-things are the intelligent agents that can perceive their internal status and external environment and can adapt themselves when they face abnormal situations [16].

The generative DBN (GDBN) model can be learned from low-dimensional data. The first objective of the model is to detect abnormalities at multiple abstraction levels (e.g., continuous and discrete levels), then incrementally learn new models from the generalized errors (GEs) coming from these anomaly data. Generalized errors (GEs) [16] can be defined as the mismatch between the Bayesian predictions and experimental evidence, computed for the hidden generalized variables of the generative model, i.e., the hidden states and the deviations of states’ together (i.e., generalized states). The higher-order derivatives are limited to first-order, and the GEs are computed through the anomaly detection process. The new model learning is a process of finding a new DBN that minimizes the presence of GEs in a given data sequence.

In the offline model incremental learning phase, the GEs produced from the sensory data sequence are used to learn the new generative model. During the online test phase, the model is tested with the datasets collected from the ego-things when they pass through novel experiences, different from the training phase. Therefore, the learned generative model can detect an abnormality as a deviation from the training experiences. Whenever the original model detects an anomaly, i.e., when the model cannot represent the current situation, the incremental learning phase can create new models dynamically. Therefore, new models can describe each new situation and will not produce any anomaly signal if a similar situation occurs in the future. Consequently, we can interpret the anomaly by explaining the differences between two models, i.e., the original and incrementally learned model. The interpretability of the models can be explained as the difference between the vocabulary of hidden variables and transition probability matrices of the different models.

### 3.1. Offline Phase: Model Learning

**Figure 1:**
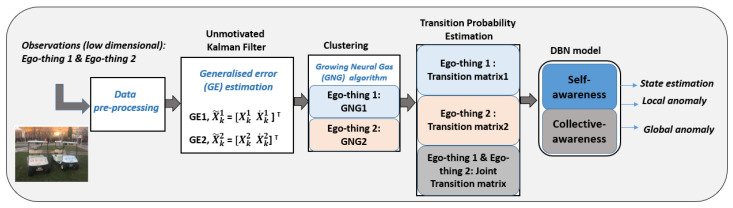
Dynamic Bayesian network (DBN) model learning process.

Figure 1 shows the block diagram of the model learning process. The total number of agents in the considered network has been limited to two and it is assumed that all the sensory data have been available to both agents in the offline and online phases. This work does not explicitly consider the communication part.

The data-driven model has different abstraction levels, such as (1) continuous and (2) discrete. The continuous level focuses on self-awareness to detect the anomaly over the filtered single agent’s signal, close to sensorial variables. This abstraction level of the model is directly related to sensory observations at the sub-symbolic level. On the other hand, the discrete level represents concepts at a symbolic level from both agents’ joint data and is dedicated to joint anomaly detection, i.e., a global anomaly. The self-awareness and collective awareness functionalities can predict the agents’ future states and detect local and global anomalies.

#### 3.1.1. Estimation of Generalized Errors (GEs)

Firstly, the data sequence collected from different agents has been synchronized to match their time stamps. This work mainly considered the proprioceptive control data of rotor velocity (*V*) to build the model. The chosen sensory data to develop the model is low dimensional, i.e., 1D vector for one agent. Let $Z_{k}^{en}$ be the measurements from the ego-thing en at the time instant *k* and $X_{k}^{en}$ be the associated latent state variable. The measured observations of ego-thing *n* can be mapped to the latent states by the following observation model:

(1) $$Z_{k}^{en}=f\left(X_{k}^{en}\right)+\delta_{k}$$

where $\delta_{k}$ represents the vector composed of measurement noise at a time step *k* and the function f() is assumed to be linear.

The application of the unmotivated Kalman filter (UKF) [17] produces GEs on the considered data sequence. This filter assumes that the state vector at time instant k+1 will be the same as the previous time instant *k*. When the observed data sequences violate this rule, generalized errors (GEs) will be generated; this is equivalent to derivatives of the data in sparse space points. In this work, we only consider GEs up to first-order derivatives. The generalized errors (GEs) related to ego-thing en can be written as:

(2) $${\tilde{X}}_{k}^{en}={\left[X_{k}\;{\dot{X}}_{k}\;\right]}^{⊺},$$

As shown in Equation (2), we have used states and their first-order derivatives as the GEs. The generalized errors are used in the next section to learn the discrete state variables of the model.

#### 3.1.2. Transition Probability from Discrete Vocabulary

The model’s discrete abstraction level has been built from the discrete vocabulary variables and different transition matrices. The clustering operation is performed separately on each ego-thing’s generalized errors (GEs).

Many clustering algorithms are available such as self organizing map (SOM) [18], K-means [19], neural gas (NG) [20], growing neural gas (GNG) [21], etc. In SOM, the total number of clusters needs to be defined before, and a wrong number may sometimes fail to represent the characteristics of data structure. In contrast to the SOM, GNG is an unsupervised incremental learning technique. GNG does not need a pre-defined number of nodes/clusters. This dynamic property of GNG is an advantage over other clustering algorithms in many applications. Considering the nature of the dataset used in this work, we selected the GNG algorithm as it has many advantages over other clustering algorithms.

The GNG algorithm separately clusters the generalized errors (GEs) that belong to each ego-thing. Each cluster is represented by its centroid/node, a 2D vector, and the centroid is a mean value of all the GEs belonging to that particular cluster. Each cluster describes a mean change w.r.t. a previous model in a given region of the state space.

The input of each GNG is a 2D vector, i.e., generalized errors of states and first-order derivatives. Therefore, one GNG is dedicated to each ego-thing. For example, the input vectors to the GNGs for the two ego-things can be written as:

(3) $$\mathrm{Ego}-\mathrm{thing}\;1:\;X_{k}^{1}={\left[v_{1}\;{\dot{v}}_{1}\right]}^{⊺}$$

(4) $$\mathrm{Ego}-\mathrm{thing}\;2:\;X_{k}^{2}={\left[v_{2}\;{\dot{v}}_{2}\right]}^{⊺}$$

For instance, the group of nodes created by a GNG of each ego-thing can be written as:

(5) $$S^{e1}=\left\{P_{1},P_{2},\cdots ,P_{m}\right\}$$

(6) $$S^{e2}=\left\{Q_{1},Q_{2},\cdots ,Q_{m}\right\}$$

where *m* represents the maximum number of nodes/clusters produced by the GNG. A word, which is formed by each unique combination of the nodes can be represented as below.

(7) $$W^{c}={\left[P_{a}\;Q_{b}\right]}^{T}$$

where $P_{a}$ represents the *a*th element of the group of nodes produced by GNG1 for ego-thing 1. Likewise, $Q_{b}$ represents the *b*th element of the list of nodes generated by GNG2 (i.e., the GNG for ego-thing 2). The next step is to estimate the transition probability matrices for each ego-thing separately and joint transition links. For example, the transition probability matrix belonging to ego-thing 1 can be written as below:

(8) $$T_{e1}=\left(\begin{array}{cccc} P_{11} & P_{12} & \cdots & P_{1n}\\ \vdots & \ddots & \\ P_{n1} & P_{n2} & \cdots & P_{nn} \end{array}\right)$$

where *n* represents the total number of nodes produced by the GNG, each element in the matrix represents the probability of transition between the discrete random variables (belonging to ego-thing 1).

Then joint transition matrix is obtained from the list of words (refer Equation (7)). This matrix represents the joint transition probability between the two ego-things and can be written as below.

(9) $$T_{joint}=\left(\begin{array}{cccc} W_{11} & W_{12} & \cdots & W_{1m}\\ \vdots & \ddots & \\ W_{m1} & W_{m2} & \cdots & W_{mm} \end{array}\right)$$

where *m* represents the total number of joint vocabulary variables (i.e., words) and each element represents the transition probability between those variables.

**Figure 2:**
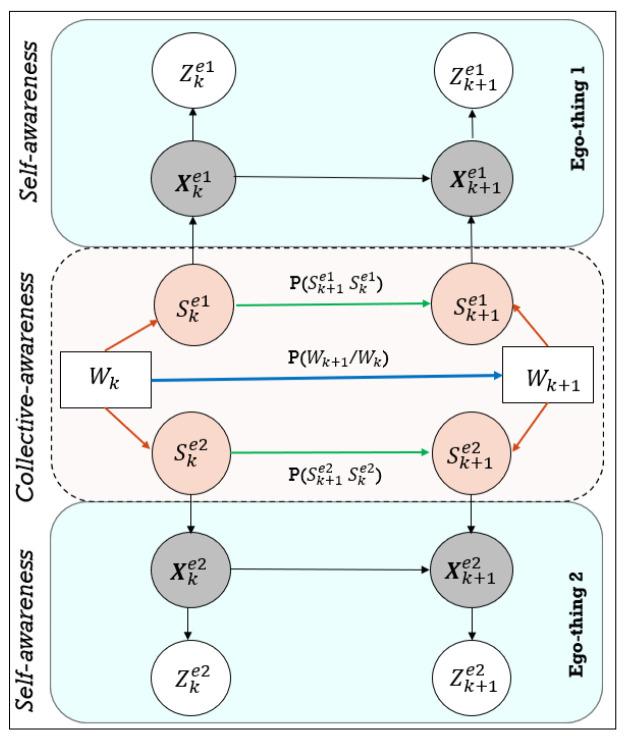
Generative dynamic Bayesian network (GDBN) model for a two ego-thing network: The cyan and orange shaded area of the model represents self-awareness and collective-awareness. Horizontal and vertical arrows represent the probabilistic connection between the variables and different abstraction levels.

The learned GDBN model representation is shown in Figure 2.

### 3.2. Online Phase: Model Testing

The model learned in the offline phase is mapped onto a linear switching model using generalized coordinates [17] that can be represented as a DBN. As proposed in [22], an inference method appropriate for such models is the distributed Markov jump particle filter (D-MJPF) that allows making joint inferences on semantic vocabulary and continuous variables composing the model. Self-awareness-oriented versions of such filters can embed extensions allowing to compute anomalies and generalized errors as part of the Bayesian reasoning performed on incoming data sequences. This makes it possible to use such filters as tools for the incremental learning process of a self-aware agent aiming to keep dynamic stability with the surrounding environment by updating models based on descriptions of changes in the forces that determine dynamics of observed patterns. Such descriptions are provided naturally by D-JMPF using generalized coordinates as variational components with respect to existing vocabulary. This makes such filters attractive for interpretability. Distributed versions of such filters can be used to embed vocabularies related to actions performed by networks of agents.

**Figure 3:**
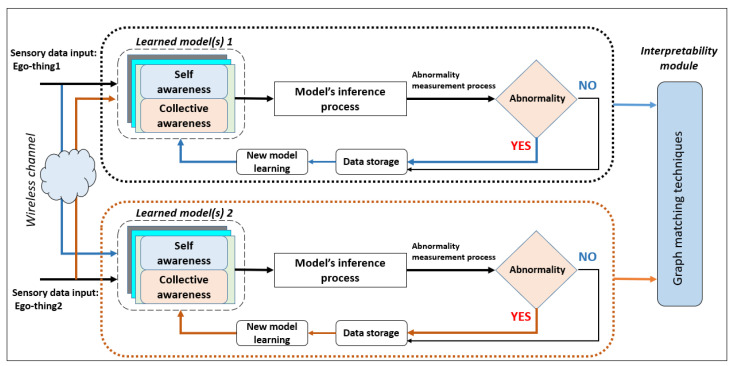
General block diagram of model testing and continual learning.

In this phase, the D-MJPF is applied to the learned generative DBN model to make inferences on the observed data. Figure 3 shows the block diagram for the model testing and continual incremental learning of new models. The model can detect global and local anomalies in the agents’ network. Global anomaly means the anomaly happens anywhere in the network, and local anomaly focuses on the anomaly around a particular agent.

#### 3.2.1. Anomaly Detection: D-MJPF

Different filters are available to solve state estimation problems, especially switching filters such as Markov jump linear system (MJLS), Rao Blackwellized particle filter, and interacting multiple model (IMM) filters to model different dynamics of agents. The MJLS is valid for linear systems with Gaussian noise and the Rao Blackwellized particle filter [23] for non-linear systems with non-Gaussian noise. Moreover, the IMM filter [24] allows an agent to predict and estimate target motion according to multiple probabilistic models. In the IMM, model switching mainly depends on a time-independent transition probability matrix. In contrast, the co-occurrence probability and transition models learned in MJPF are time-dependent, allowing a time-variant transition probability specific for each dynamic model. In this work, we used MJPF, a type of MJLS as the system considered in this work is linear and noise is Gaussian. Moreover, the transitions models learned are time-dependent.

The MJPF [25] is a hybrid filter: a Kalman filter at the continuous level and particle filter at the discrete level to make inferences. A detailed description of the MJPF can be found in [16,22]. However, in this paper, we have used a modified version of it by adding the functionality to detect discrete level global anomaly along with the local anomaly detection capability.

The D-MJPF designed in this work is generative, and the posterior probability density function can be written as:

(10) $$p\left(W_{k+1},{\tilde{X}}_{k+1}/Z_{k+1}\right)=p\left({\tilde{X}}_{k+1}/W_{k+1},Z_{k+1}\right)p\left(W_{k+1}/Z_{k+1}\right)$$

where $W_{k+1}$ represents the word in the discrete level and ${\tilde{X}}_{k+1}$ is the continuous state in the state space at time instant k+1. $p(W_{k+1}/Z_{k+1})$ is estimated by using particle filter [26] at the discrete vocabulary level. The predictions $p({\tilde{X}}_{k+1}/{\tilde{X}}_{k})$ of particles at the continuous state level are obtained by considering a bank of Kalman filters built according to the discrete vocabulary where different Gaussian velocity models are valid for each switching variable.

The inference starts with an initial observed random data point. It then estimates the discrete node by calculating and selecting the cluster nodes with a minimum distance with the ground truth observation. The selection of a control vector for the future state prediction by the continuous level part (i.e., Kalman filter) is influenced by the probability of discrete level variable estimated before. The prediction at the continuous level is made separately for both agents, which is part of the model’s self-awareness functionality. The particle filter at the discrete level makes the inference of joint state prediction and global abnormality estimation. Moreover, the system can continually learn new models whenever the ego-things pass through new experiences.

- Local anomaly detection: Self-awareness
  The continuous level of the learned DBN model (refer to Figure 2) is dedicated to predicting future states of ego-things separately and detecting anomalies around a specific ego-thing. The *innovation* measurement of the D-MJPF is used and can be estimated as below:
  (11) $$\theta_{k,e1}=Z_{k}^{\left(e1\right)}-HX_{k}^{\left(e1\right)}$$
  (12) $$\theta_{k,e2}=Z_{k}^{\left(e2\right)}-HX_{k}^{\left(e2\right)}$$
  where $\theta_{k}$ represents the innovation terms, the $Z_{k}$ term represents the ground truth sensory observation, and $X_{k}$ is the predicted state.
  The innovation measurements (i.e., abnormality signal) produced (see Equations (11) and (12)) from the anomaly detection process is used to learn new models incrementally and to represent the situation. Once detected anomaly at continuous level (i.e., local abnormality), the GEs produced from the anomaly signal have been used to correct the model incrementally. Here the GEs are the predicted states and the anomaly measurements (i.e., the probabilistic distance between predicted states and observed evidence). The new model has to capture the current situation that produced anomalies and predict the states better when similar situations occur in the future.
  The GEs signal of the anomaly has been firstly clustered by applying the GNG algorithm. These clusters generated from the GEs will mainly differ from previously generated clusters on those state-space regions where the anomaly occurred. Then the information extracted by the clustering is used to learn vocabulary and transition probability matrix. The transition probability (shown as green arrows in Figure 2), which influences the model’s continuous level, will be improved to predict the situation well and consequently to produce low or null GEs.
- Global anomaly detection: Collective awareness
  The DBN models’ discrete level (shaded in orange color) shown in Figure 2 detects the global anomaly, i.e., the anomaly happening anywhere in the network. In co-operative task scenarios, this metric can detect the anomaly happening around other agents in the network. The metric used to estimate anomaly is Kullback–Leibler divergence [27]. The Kullback–Leibler (KL) divergence measures how a probability distribution differs from another probability distribution. This work has used this metric to estimate the difference between the predicted discrete level variables distribution and the discrete state’s distribution estimated from the observed sensory variables. All the variables considered here in the discrete level are calculated jointly by considering both ego-things. The equation below calculates the KL distance:
  (13) $$D_{\mathrm{KL}}\left(\lambda \|\pi \right)=\sum_{x\in X}\pi \left(x\right)\log\left(\frac{\lambda (x)}{\pi (x)}\right)$$
  where π is the joint distribution of predicted discrete random variables and λ is the joint distribution of observed discrete state variables.
  Once a global abnormality is detected, i.e., an anomaly happens anywhere in the ego-thing’s network, firstly list out those ego-things that encountered abnormal situations locally. Then the GEs of the abnormality signals detected by the ego-things at the continuous level will be clustered separately. This clustered information will be used to update the corresponding discrete vocabulary and transition probability matrices. The very next step is to update the joint vocabulary (words) and joint transition probability matrix (represented by the *green* arrow in Figure 2). Therefore, if a similar joint event occurs in the future, the model can represent the situation to make appropriate decisions.

#### 3.2.2. Continual Model Learning and Interpretability

Model learning is a continuous process whenever the existing models cannot represent the ego-thing’s current experience, i.e., when the model detects an abnormality. The developed ML model’s interpretability focuses on the model’s intermediate results, such as discrete level vocabulary and transition probability matrices.

The interpretability module (refer to Figure 3) clarifies the reasoning behind the model’s decision-making under different circumstances. In this work, the term interpretability can be defined as the capability of the model to detect the anomaly and use this information to learn the model incrementally. This work mainly focused on model-based interpretability, i.e., the development of models that readily give insight into the relationships they have learned [6] and exploited graph matching [28] techniques to explain the interpretability. Graph matching techniques were performed to compare the graphs such as semantic vocabularies produced at different stages of model testing phases w.r.t. to graphs generated of the initial model learning phase. As described previously, the model makes inferences at different abstraction levels, such as continuous and discrete levels, and examples have been explained in Section 5.

In the initial model learning phase, vocabularies and transition probability matrices are learned for each ego-thing separately and collectively. Each element of the transition matrices can be seen as a link between two nodes. Nodes are elements of the vocabulary. Nodes and edges can represent the graph formed from the vocabulary as below.

(14) $$\mathrm{Ego}-\mathrm{thing}\;1,\;G_{e1}=\left(S1,E1\right)$$

(15) $$\mathrm{Ego}-\mathrm{thing}\;2,\;G_{e2}=\left(S2,E2\right)$$

where Sn and En represent the number of nodes and edges, respectively. Each unique combination of the nodes belonging to ego-thing 1 and ego-thing 2 form a word (refer Equation (7)), and a dual graph can represent such a list of words (refer Section 5.2). The transition probability matrices belong to each ego-thing separately as well as collectively are represented by Equations (8) and (9), respectively.

Comparing two vocabularies and related transition matrices represented as graphs allows explaining differences caused by anomalies in the agents’ network. In this work, we perform inexact graph matching [29], i.e., the number of nodes is different in both graphs. We assume the number of nodes in the source graph will be less than the one in the target graph, i.e., |S1|<|S2|. The aim is to find a mapping $f^{{}^{\prime }}$: S1→S2. It means a search for a small graph within a big graph. The same operation is being performed for transition probability matrices as well. The rows and columns of the transition probability matrices have been compared to find the match. The mismatching part of the vocabularies (i.e., cluster) and transition probability matrices indicate the presence of an anomaly.

The initial model has been learned by following the steps shown in Figure 1. The graphs such as transition probability matrices for each ego-thing, dual graphs, and joint transition matrices generated at intermediate steps of model learning have been stored. This initial model tests with datasets collected from different scenarios. When the model detects the abnormality, incremental learning of the new model will be performed to minimize the GEs. The graphs generated at each intermediate stage of this incremental learning phase can be compared with previously learned models. The differences obtained from the comparison are due to the abnormal situation in the ego-thing network.

The model’s continuous level focuses on self-awareness to detect local anomalies. By testing the model with different scenario datasets, the system extracts knowledge of abnormalities and learns new models incrementally by exploiting the anomaly data. As stated before, the continuous level of the model is concerned about the individual ego-thing’s experience. The continuous level’s limitation is that it does not take into account the experience of other ego-things in the network. The discrete level emphasizing collective awareness and global anomaly detection will help overcome the limitations of the continuous abstraction level.

In the MJPF, each dynamic model in the continuous state is associated with one of the vocabulary variables at the discrete level. During the anomaly interval (when a pedestrian crosses in front of the iCab vehicles), the continuous states predicted by the model (with the chosen discrete variable’s influence) will be different from the ground truth observed data. Such a difference is called GEs or abnormality and is used to learn new models incrementally. The generative DBN model’s interpretability can be explained with the GEs (i.e., anomaly), discrete vocabulary, and transition probability matrices.

Once the model detects a local abnormality, the GE data are stored (refer to Figure 3) and use this data to learn a new model incrementally by following the steps shown in Figure 1. The main changes in the new model w.r.t. the previous model will be in the clusters of GEs, vocabulary, and the transition probability (*green* arrows in Figure 2) between the discrete level vocabulary variables.

Whenever the ego-things experience a new situation that was not seen in the past, the system extracts knowledge and updates/learns a new model to represent the new situation. So that if we consider a particular stage of the filter, it will be able to describe all the previous experiences passed by the ego-things. If similar situations occur in the future, the models would easily represent them and help take appropriate decisions.

Contrary to the model’s continuous level (sub-symbolic level) that can detect a local anomaly, the model’s discrete level (symbolic level) will mainly focus on global anomaly detection, i.e., the abnormality around any ego-thing in the network. The interpretability feature supports the continual learning of new models when the system passes through new joint experiences. With this collective awareness functionality, the model inside one ego-thing can detect other ego-thing anomalies in the network. It would help the model to make appropriate decisions in different situations. Any new experience of individual ego-things will affect both the continuous and discrete levels and, consequently, update the relevant part of the model. However, the collective experiences of the ego-things may not necessarily make updates at the continuous abstraction level of the model.

## 4. Experimental Study

**Figure 4:**
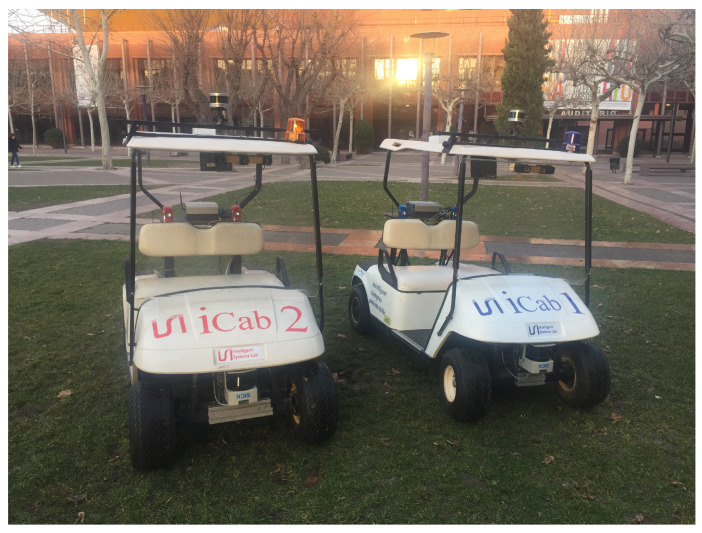
iCab vehicles.

This section experimentally validates the proposed methodology of learning and testing interpretable machine learning models. For the case study, we have considered and small network of two intelligent autonomous vehicles named *iCab* (intelligent campus automobile) having the same setup [30] and shown in Figure 4. The multi-sensory low-dimensional datasets collected from the cooperative driving tasks are used to learn and test the model. The number of ego-things in the network can be increased to use in safety and security applications in cooperative driving in intelligent transportation systems. The methodology can generally be applied in any agent network performing collaborative tasks. However, it needs modifications in the models and embeds efficient communication schemes.

**Figure 5:**
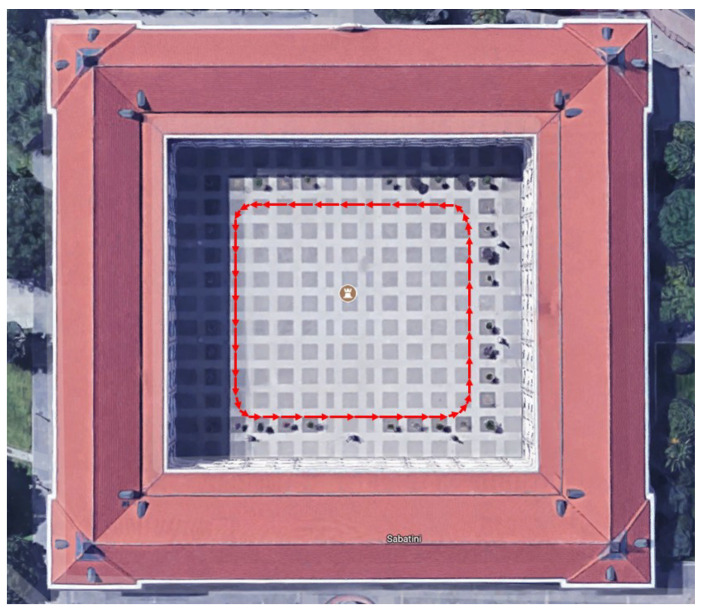
Testing environment (38 m × 33 m).

**Figure 6:**
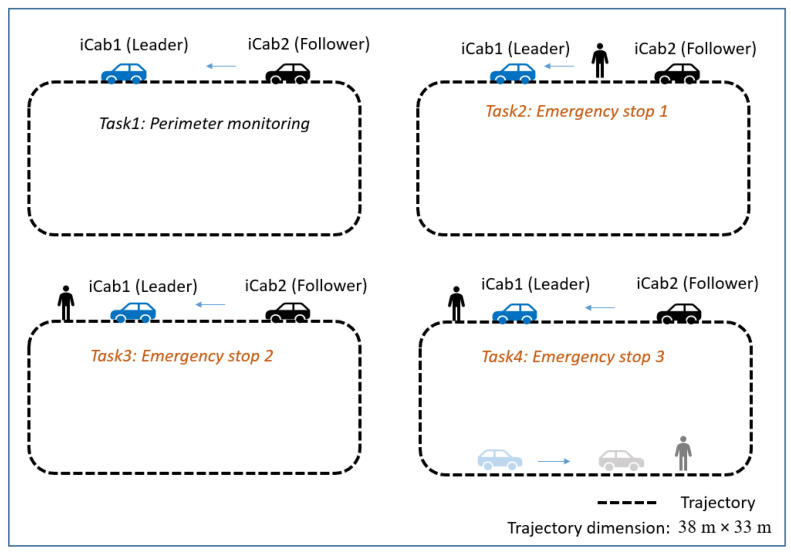
Co-operative driving tasks: The different co-operative driving scenarios considered. The datasets from Task 1 were used in the training phase to learn the model, and the remaining tasks (Task 2 to Task 4) were used in the test phase to check the model’s fitness in detecting local and global abnormality.

The vehicles in the small network are equipped with sensors, such as one LIDAR, a stereo camera, laser rangefinder, and encoders. This work focused on the vehicles’ low-dimensional control data, i.e., rotor velocity (*v*). The collected data from both vehicles are synchronized to align their timestamps. The two iCab vehicles perform joint navigation tasks in the rectangular trajectory shown in Figure 5 by keeping their position one after the other with a minimum distance among them. The vehicle navigating in the front is called the *leader* (*iCab1*) and the one follows is the *follower* (*iCab2*). The different experiments conducted to collect the datasets are shown in Figure 6.

### 4.1. Training Datasets

Perimeter monitoring (Task 1): the datasets are collected while the vehicles jointly perform platooning operation in a closed environment, as shown in Figure 6: Task 1 is used in the training phase to learn the model. The navigation operation was performed four times, one after the other, and corresponding data have been collected. The *follower* vehicle (*iCab2*) mimics the actions of the *leader* (*iCab1*) vehicle. The dataset considered for the model development is composed of the vehicles’ rotor velocity (*V*) while they perform joint navigation tasks.

### 4.2. Test Datasets

**Figure 7:**
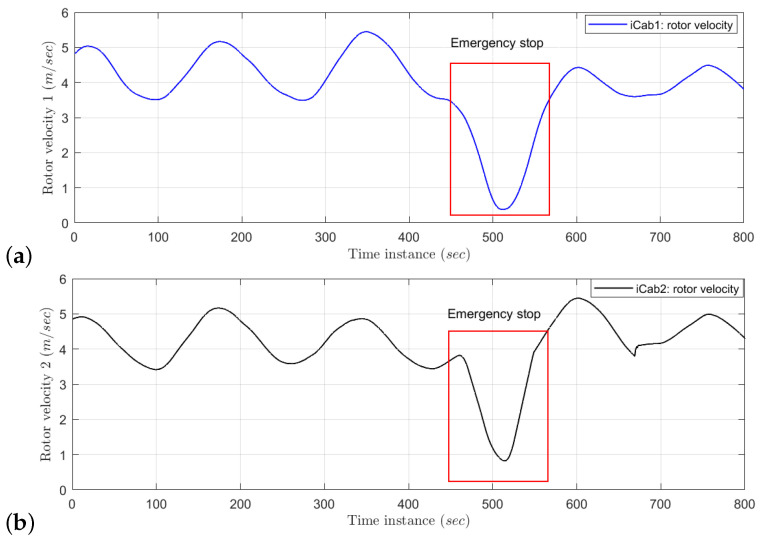
Test data: Emergency stop. (**a**) *iCab1* rotor velocity, (**b**) *iCab2* rotor velocity. A total of four data samples are collected at every second.

The example of rotor velocity test data for *iCab1* and *iCab2* when performs joint navigation operation of Task 3 is plotted in Figure 7. During the emergency brake operation (when the vehicle encounters a pedestrian), the velocity values are reduced, if compared to the training data sequence and are marked by the red rectangular box. The rotor velocity data (*V*) of both vehicles while performing the below tasks have been collected and used in the learned model’s test phase.

1. Emergency stop 1 (Task 2): while both vehicles jointly navigate a rectangular trajectory, an unexpected pedestrian appears in front of the follower vehicle (*iCab2*), which performs an emergency brake operation. Since the leader vehicle is not encountering any obstacles, it continues the navigation task. The follower vehicle continues the navigation once the pedestrian crosses the danger zone.
2. Emergency stop 2 (Task 3): the task is similar to the previous one (Emergency stop 1), except the pedestrian appears in front of the leader vehicle (*iCab1*). As soon the leader detects the presence of the pedestrian, it performs an emergency brake operation. Subsequently the follower (*iCab2*) vehicle also performs emergency brake. Once the pedestrian crosses, both the vehicles (*iCab1* and *iCab2*) continue the joint perimeter monitoring task.
3. Emergency stop 3 (Task 4): the task is similar to previous scenarios, but in this case, pedestrians appear in two locations in the trajectory.

## 5. Results and Discussion

The anomaly detection results estimated by the D-MJPF at different abstraction levels, such as continuous and discrete levels, are discussed in this section. The self-awareness functionality is embedded in the model’s continuous level to detect the local abnormality. On the other hand, the discrete level models the collective awareness functionality to detect a global abnormality.

### 5.1. Abnormality Detection by D-MJPF

**Figure 8:**
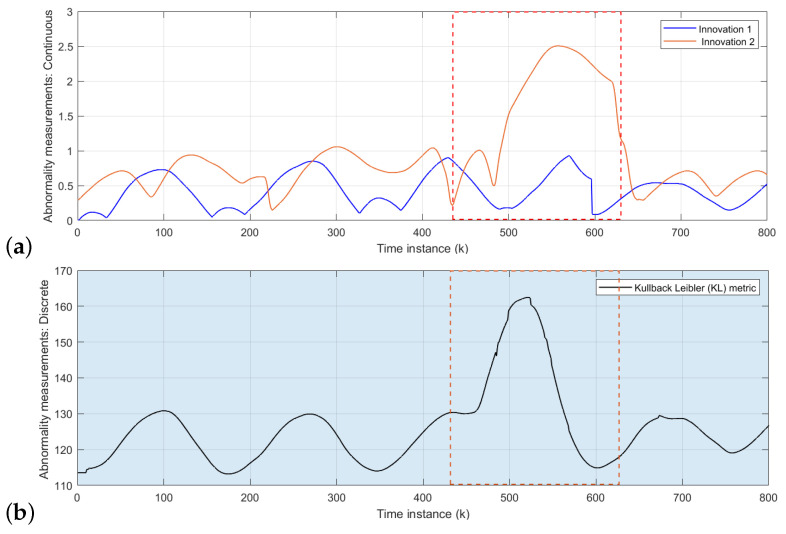
Abnormality measurements: Task 2 test data: (**a**) Continuous level anomaly: the blue and orange plots belong to the anomaly estimated for *iCab1* and *iCab2*, respectively. The highest peaks of the orange plot represent the situation where the *iCab2* performed an emergency stop operation when it detected a pedestrian’s presence. (**b**) Discrete level abnormality: the peaks show the global anomaly detected by the model.

**Figure 9:**
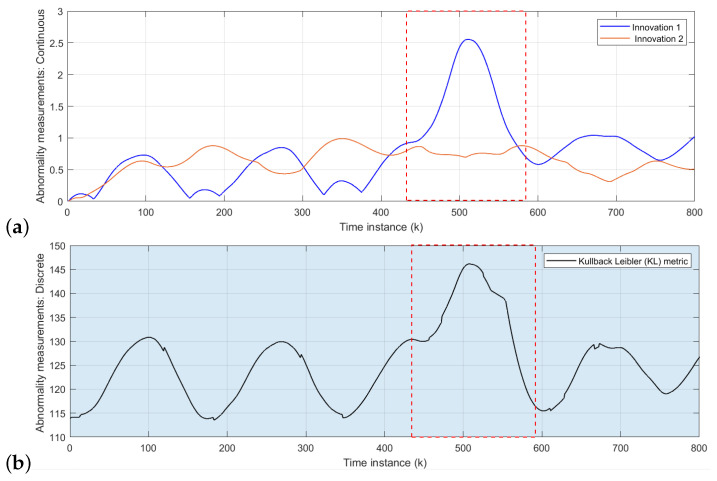
Abnormality measurements: Task 3 test data: (**a**) Continuous level anomaly: the blue and orange plots belong to the anomaly estimated for *iCab1* and *iCab2*, respectively. The blue plot’s highest peaks represent the situation where the *iCab1* performed an emergency stop operation when it detected the presence of a pedestrian. (**b**) Discrete level abnormality: the peaks show the global anomaly detected by the model.

**Figure 10:**
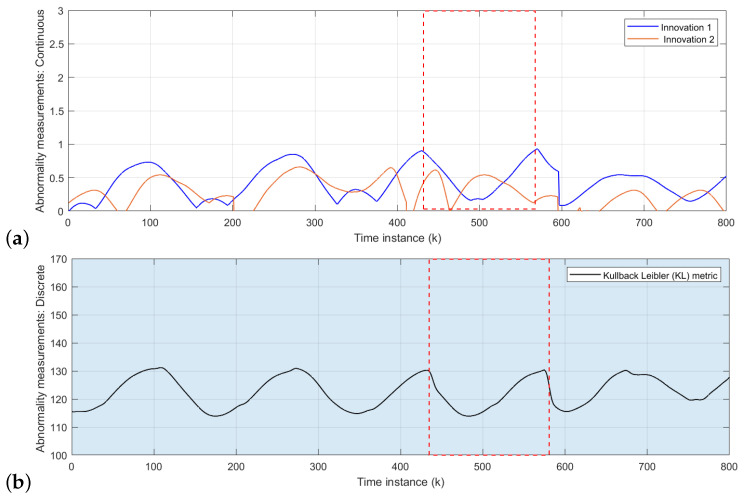
Abnormality measurements: Task 4 test data: (**a**): the blue and orange plots belong to the anomaly estimated for *iCab1* and *iCab2*, respectively. (**b**) Discrete level abnormality: the peaks show the global anomaly detected by the model.

The area inside red dashed box from Figure 8, Figure 9 and Figure 10 indicates the time interval of abnormal situations.

#### 5.1.1. Local Abnormality Detection: Self-Awareness

The initial model is tested with the rotor velocity datasets collected from Task 2 (refer to Figure 6) joint navigation of vehicles. The continuous level part of the D-MJPF shown in Figure 2 is dedicated to the self-awareness of the ego-things to predict future states and detect a local abnormality, i.e., the anomaly that happens around an individual ego-thing. The anomalies estimated by the innovation metric of the filter when tested with different datasets are plotted in Figure 8a, Figure 9a and Figure 10a.

Figure 8a shows the abnormality signal obtained when the initial model M0 (learned from Task 1 in Figure 6) is tested with the sensory data sequence of Task 2. Since a random pedestrian appears in front of the *iCab2* vehicle, it performs an emergency brake; however, iCab1 continues the perimeter monitoring task without any interruption. Therefore the local anomaly detected by the model is only for the *iCab2* vehicle shown by the peak of *orange* plot inside the marked region. In this situation, a new model ($M_{1}$) that minimizes the GEs will learn incrementally to represent the new situation in which this incremental model learning phase is enabled.

In the next step, the cooperative driving Task 3 (Emergency stop 2) scenario dataset is used to test the model $M_{1}$. The resulting anomaly signals are shown in Figure 9a. The blue and orange plots indicate the anomaly for *iCab1* and *iCab2*, respectively. In the Task 3 scenario, the pedestrian appears in front of the leader (*iCab1*) vehicle, and both vehicles perform emergency stop operations. Although both vehicles stopped with a pedestrian’s presence, the anomaly is detected for *iCab1* only. This is because *iCab2* already experienced a similar situation before, and the model was able to represent the current situation. Therefore, the incremental learning update was only for the *iCab1* and learned a new model called $M_{2}$.

As a final test phase, the model $M_{2}$ has been tested with another cooperative scenario dataset of Task 4 in Figure 6. The model-generated anomaly signals are plotted in Figure 10a. As expected, the model could represent the situation even if the pedestrian appeared in two spatial locations of the joint navigation task’s trajectory. Because the existing model has previously experienced a similar situation, there were not any higher peaks in the anomaly signals. The model is learned from proprioceptive control data of rotor velocity (*V*) so that the models’ performance is independent of spatial locations.

#### 5.1.2. Global Abnormality Detection: Collective-Awareness

The global anomaly detection part belongs to the inference made by the discrete part of D-MJPF (orange shaded area in Figure 2). The model can detect the global anomaly that happens anywhere in the network. The global anomaly has been plotted in Figure 8b, Figure 9b and Figure 10b. The different test phases of the model are the same as explained in the previous section (Section 5.1.1).

At first, the initial model (M0) was tested with the dataset collected from Task 2, shown in Figure 6. The peaks in Figure 8b indicates the presence of pedestrian appearing in front of the *iCab2* vehicle when tested with the velocity dataset of Task 2 (refer to Figure 6). The discrete level part of the model is updated after detecting the anomaly, and the new learned model is called $M_{1}$. In the next step, we tested the model ($M_{1}$) with another dataset from Task 3 in Figure 6. This time the pedestrian appears in front of the leader vehicle, and an emergency stop operation is performed by both vehicles (*iCab1* and *iCab2*). Therefore, the interaction among the vehicles is slightly different from the one learned in the previous step. As a result, the model detected an anomaly and is plotted in Figure 9b, and the model will be updated to the next version and is called $M_{2}$.

Finally, the model $M_{2}$ was tested with the data of Task 4 (refer to Figure 6), and the resulting anomaly is plotted in Figure 10b. There are not any higher peaks this time as the model could represent this situation with the existing knowledge learned previously.

### 5.2. Interpretability and Comparative Analysis

The detection of anomalies in a particular time range can be interpreted when analyzed as part of the incremental learning process. Generalized errors produced during the anomaly detection step can be used to describe the causes of the detected anomalies in terms of variations induced in the vocabulary of the filter. This can be explained based on the free energy minimization principle [31]. The new optimal learned model will produce minimum generalized errors to minimize free energy on the sequence that produced anomalies. Thus, the variations in its vocabulary can be considered as an explanation of the causes that produced such anomalies. On this basis, it is necessary to use techniques to compare vocabularies of the filter that produced anomalies with respect to the one that would have minimized anomalies themselves (in a statistical sense) over the sequence. Graph matching techniques can be used to this end.

An initial model called ($M_{0}$) will be learned in the model training phase. Then each test phase incrementally learns new models ($M_{n}$, where *n* is the index of the models) whenever the existing model detects an anomaly. Each incrementally learned model will minimize the energy and represent the situation in which the model learned. The intermediate results of the models have been presented and made a comparative analysis in this section.

**Figure 11:**
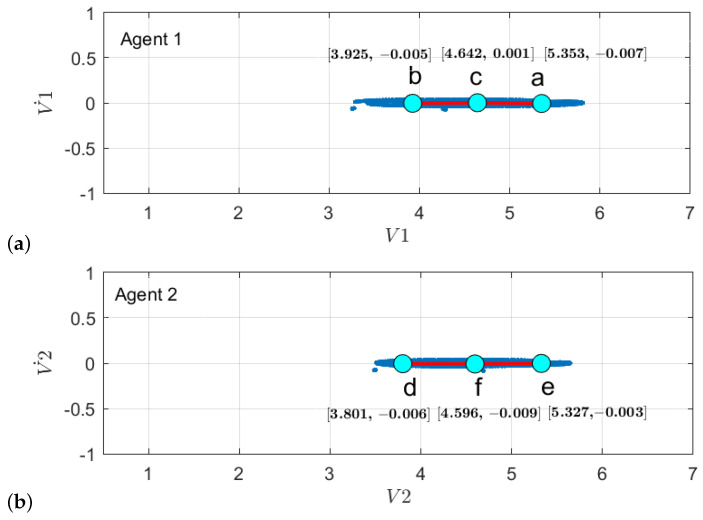
Clustering of GEs produced by applying unmotivated Kalman filter on the training data for (**a**) *agent 1* and (**b**) *agent 2*.

**Figure 12:**
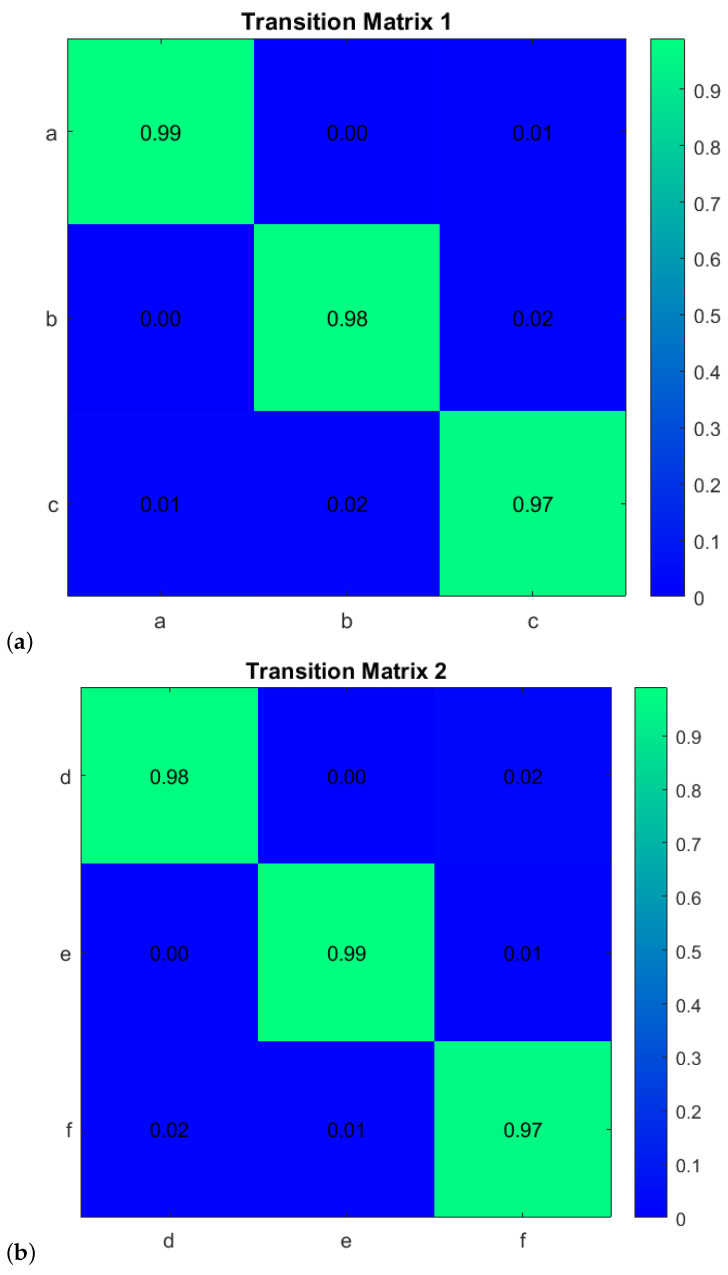
Transition probability matrices obtained from the initial model (training phase) for (**a**) *agent 1* and (**b**) *agent 2*.

**Figure 13:**
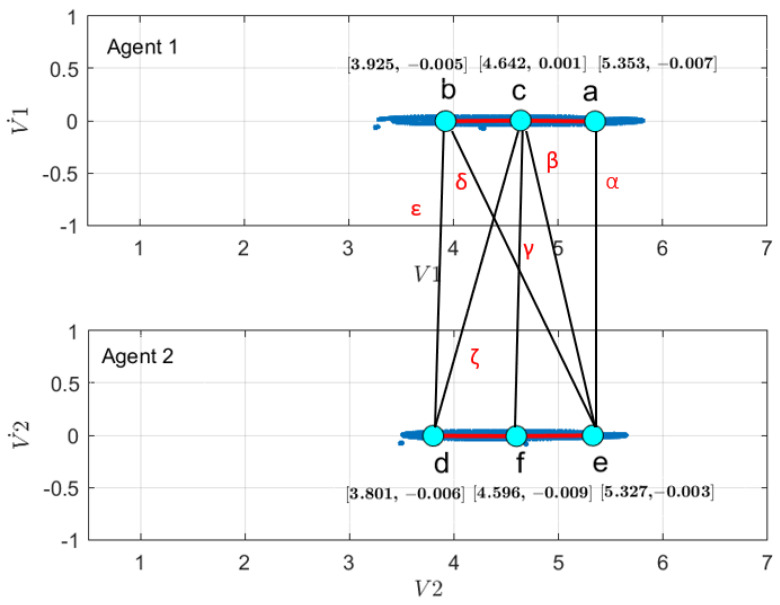
Joint vocabulary from the initial model corresponds to the coupled Bayesian network in the model (orange shaded area in Figure 2).

First, to learn the initial model (M0), estimate the GEs by applying an unmotivated Kalman filter on the training data. Then the produced GEs have been clustered by applying the GNG algorithm is plotted in Figure 11. The whole process of vocabulary and transition probability learning is detailed in Section 3.1.2. The cyan nodes represent the centroid of the clusters. The edges in red represent the transition probability between the nodes. Moreover, the transition probability matrices for each ego-thing are shown in Figure 12a,b. The estimated joint transition probability by grouping the jointly activated node pairs is shown in Figure 13. Each unique couple of activated node pairs has been represented by the black edges and named by letters in red. The activated node pairs, unique labels, and 4D vector representing each label are summarized in Table 1.

**Figure 14:**
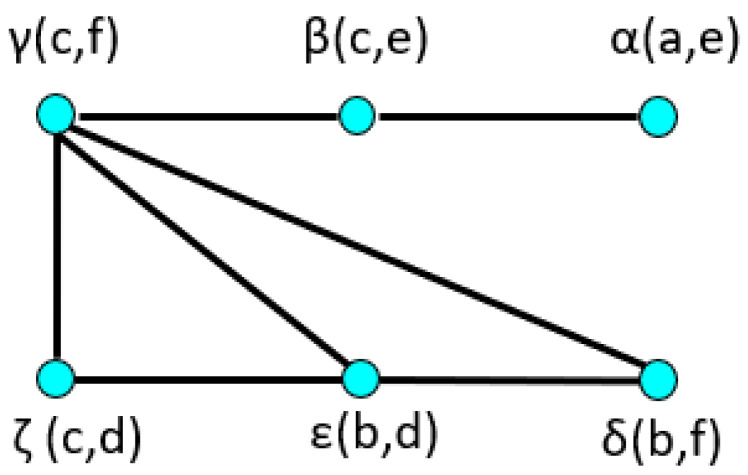
Dual graph representation of joint vocabulary and the transition probabilities of the initial model.

**Figure 15:**
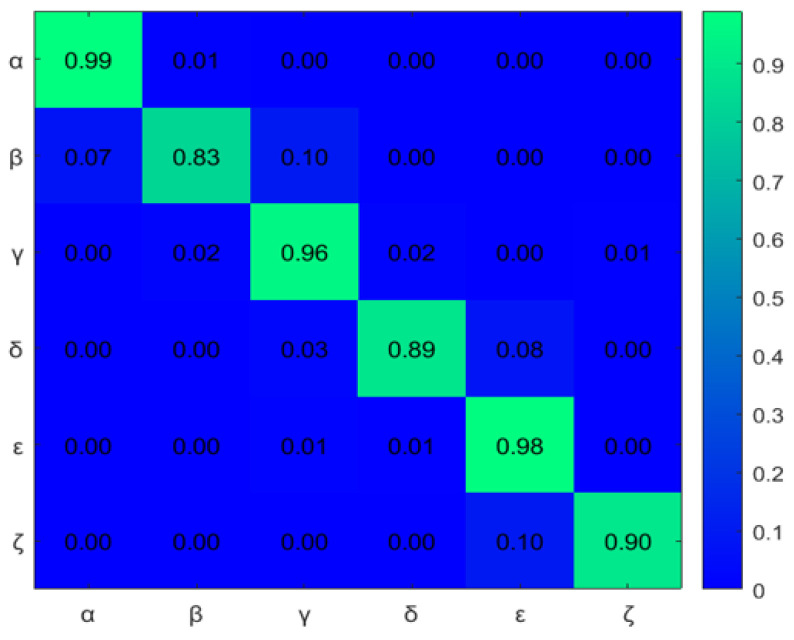
Joint transition probability matrix from the initial model.

A dual graph representation of the activated node pairs is shown in Figure 14. Figure 15 represents the joint transition probability matrix between the two ego-things. The interpretability of the model’s decision at different test phases is explained below by comparing the graphs produced at the intermediate levels with respect to the initial model.

The model’s continuous level focuses on detecting local anomalies by exploiting self-awareness functionality. On the other hand, the discrete level, i.e., collective awareness part of the model, gives attention to global anomaly detection, i.e., the abnormality around any ego-thing in the network.

#### 5.2.1. Continuous Level

**Figure 16:**
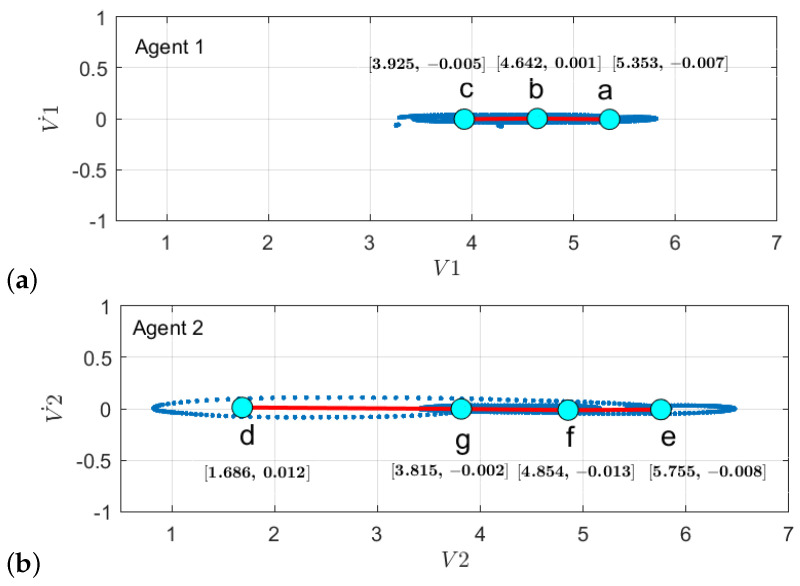
Clustering of GEs from incrementally learned model $M_{1}$ for (**a**) *agent 1* and (**b**) *agent 2*.

Figure 6 shows the experimental scenarios/tasks used in this work. The example of interpretability feature focuses on incrementally learning new models from the GEs of the anomaly signal has been explained below:

(1) When the initial model $M_{0}$ was tested with the data sequences collected from Task 2, the model detected abnormality only for *iCab2* at the continuous level and is plotted in Figure 8a. A new model $M_{1}$ will learn in this situation starting from the GEs of abnormality signal plotted in Figure 16a,b.

**Figure 17:**
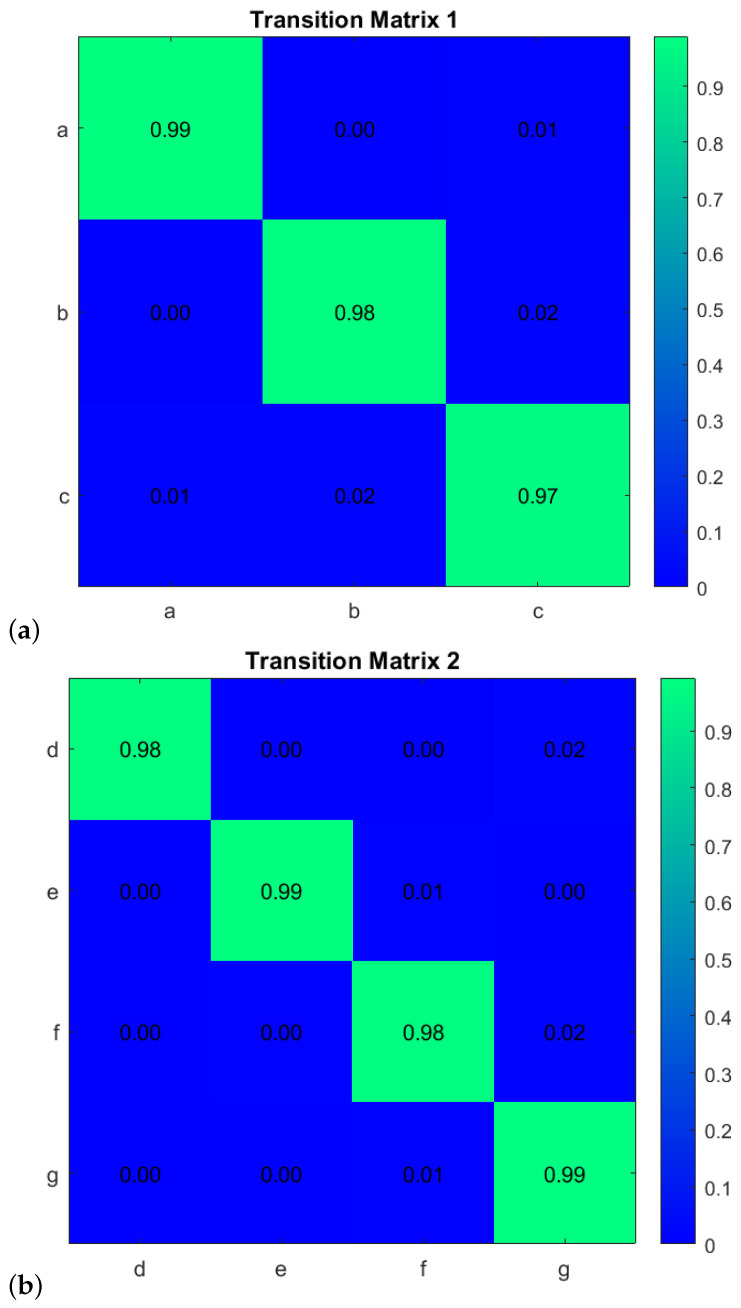
Transition probability matrices obtained from the model $M_{1}$ for (**a**) *agent 1* (**b**) *agent 2*.

With respect to the clusters produced by the initial model M0 learning phase as shown in Figure 11a, there is an additional discrete space formed which belongs to node *d* in Figure 16b when there is a cluster of GEs of a detected anomaly, causing it to learn a new model $M_{1}$. The node *d* encoded the information related to emergency stop operation when the pedestrian appeared. Subsequently, the changes, such as an increase in the number of transitions between the superstate variables w.r.t. initial model, are shown in Figure 17b. As ego-thing 1 does not encounter any anomaly, the transition probability matrix remains unchanged as shown in Figure 17a.

**Figure 18:**
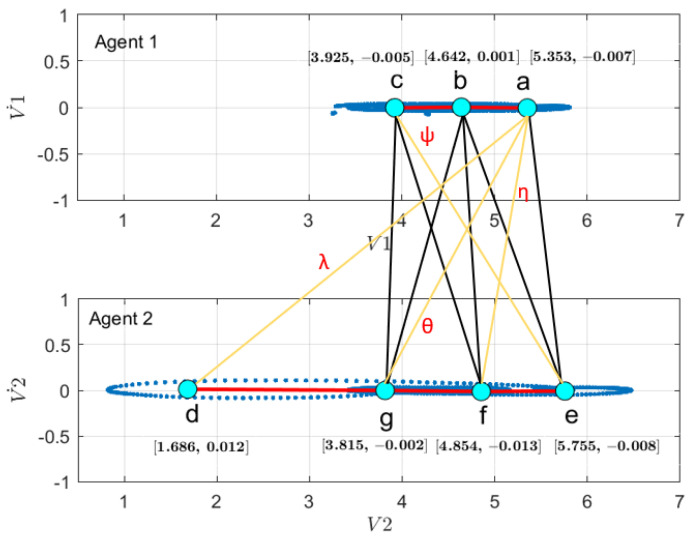
Joint vocabulary from the model $M_{1}$.

The joint vocabulary of model $M_{1}$ is represented in Figure 18. The edges shown in black are the ones that could find mapping with the graphs produced of the initial model ($M_{0}$). The orange edges are newly generated and belong to the detected anomaly. Each of the edges is given a unique label and is summarized in Table 2. The node pairs represented by red in column 3 of Table 2 belong to the anomaly.

**Figure 19:**
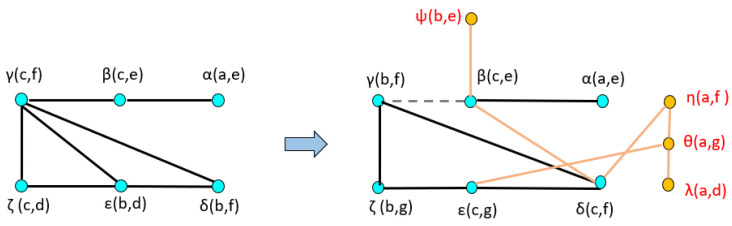
Dual graph representation and comparison of joint vocabulary between initial model $M_{0}$ and model $M_{1}$.

**Figure 20:**
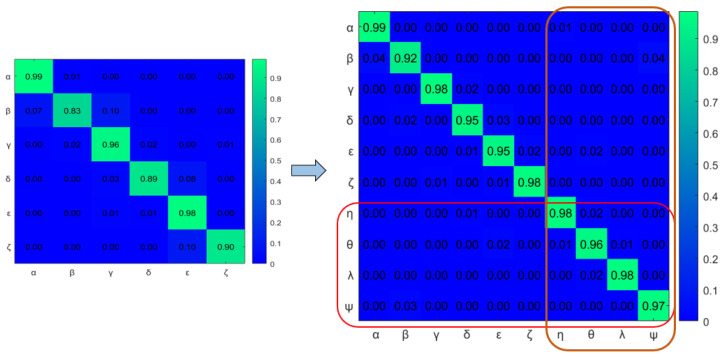
Comparison of joint transition probability matrices between incrementally learned model $M_{1}$ and initial model ($M_{0}$).

Figure 19 shows the comparison of dual graphs produced from the joint vocabulary of initial model $M_{0}$ and incrementally learned model $M_{1}$. The additionally enabled nodes and edges are represented in orange. Finally, mapping has been performed between the joint transition matrices and is shown in Figure 20. The part inside the rectangular box shows the additional transitions that occurred due to an abnormal situation w.r.t. the initial model.

**Figure 21:**
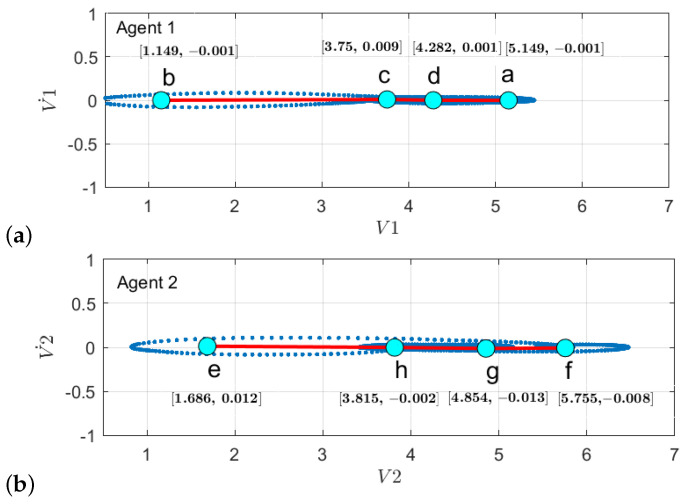
Clustering of GEs from incrementally learned model $M_{2}$ for (**a**) *agent 1* and (**b**) *agent 2*.

2. Next, the model $M_{1}$ was tested with the rotor velocity data sequence collected from Task 3. This time, the model detecting an anomaly at the continuous level belongs to *iCab1* only, and it is plotted in Figure 9a. A new model called $M_{2}$ will be learned incrementally in this phase. Since both vehicles perform emergency stop operations, the anomaly was detected only for the leader vehicle (iCab) because the follower (*iCab2*) previously experienced a similar situation. Hence, the model was able to represent well the behaviors of *iCab2*, and the model correction was required only for the part related to *iCab1*. The GEs produced from the anomaly data for *iCab1* and *iCab2* are plotted in Figure 21a and Figure 21b, respectively. The additional node generated is for *iCab2* only as iCab 1 experiences similar situation while learning the model $M_{1}$. The parameters extracted from the clusters are used to generate discrete vocabulary and transition probability matrix to learn the model $M_{2}$.

**Figure 22:**
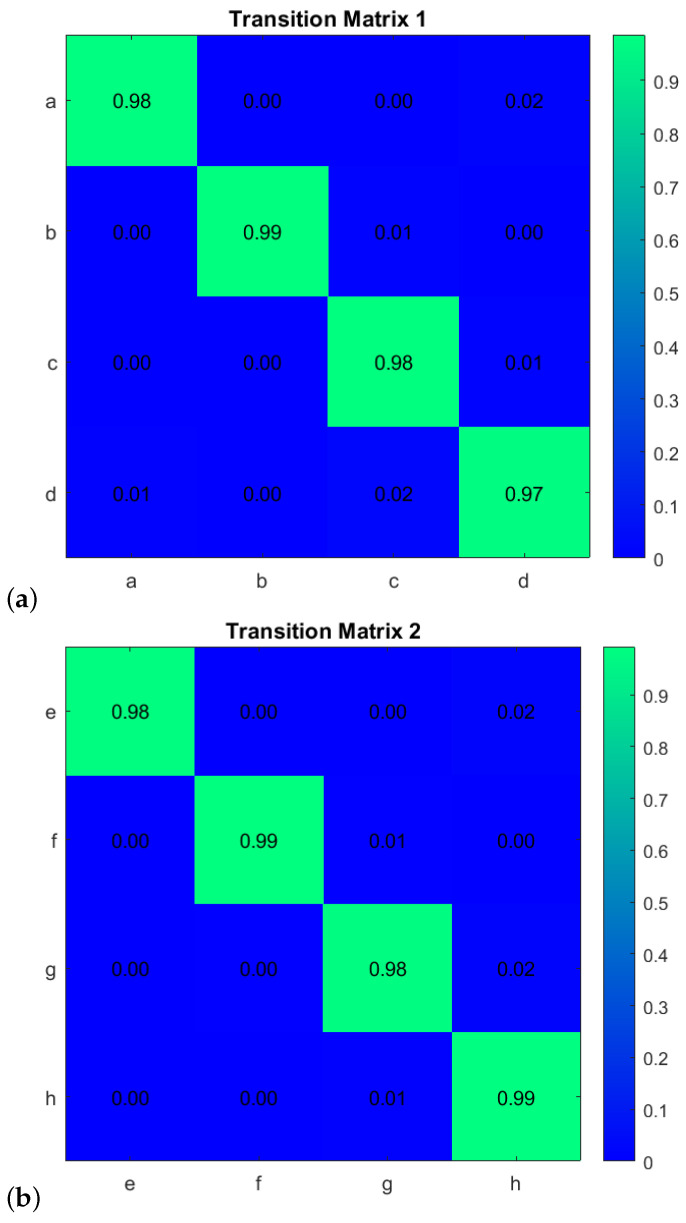
Transition probability matrices obtained from the model $M_{2}$ for (**a**) *agent 1* and (**b**) *agent 2*.

**Figure 23:**
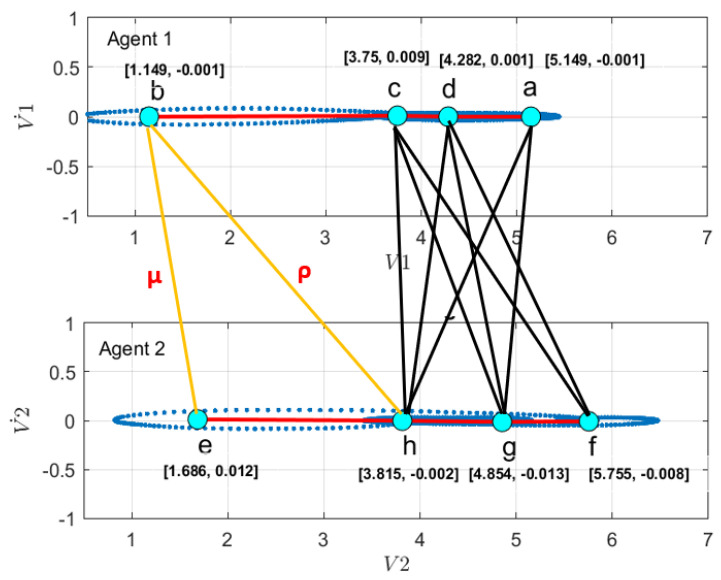
Joint vocabulary from the model $M_{2}$.

**Figure 24:**
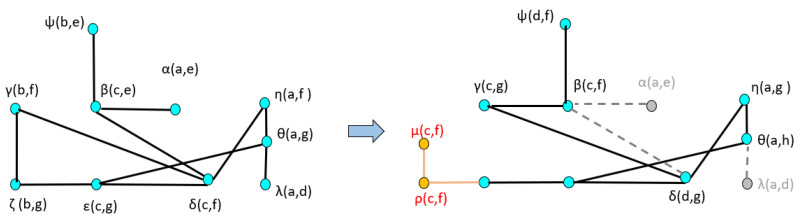
Dual graph representation and comparison of joint vocabulary between model $M_{1}$ and model $M_{2}$.

**Figure 25:**
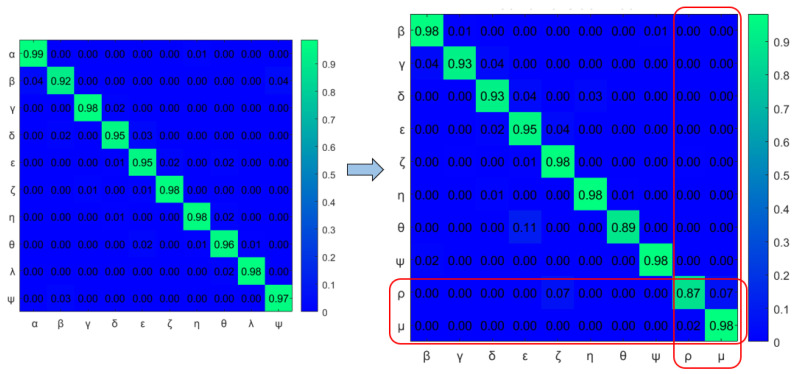
Comparison of joint transition probability matrices between incrementally learned model $M_{2}$ and previously learned models.

The transition probability matrix for ego-thing 1 has been updated as shown in Figure 22a. The joint vocabulary is represented in Figure 23, two additional edges such as μ and ρ generated w.r.t. the previous cases encode the presence of an anomaly. Figure 24 and Figure 25 show the comparison of dual graph representation and the joint transition probabilities, respectively.

3. Finally, we tested the model $M_{2}$ with the data collected from Task 4. This time, a random pedestrian appeared two times in the entire trajectory of the vehicles’ maneuvering operation. However, the model has not detected anomaly at the continuous level (see Figure 10a) because the existing model was able to represent well the current situation and to predict correctly the states of the vehicles. Therefore, matching the dual maps and joint transition probability matrices between Task 3 test data w.r.t. to the previous case did not produce any error. Thus, the model remains unchanged as the existing model could represent the current situation.

#### 5.2.2. Discrete Level

The interpretability can be visible by checking the intermediate results. The process of continual learning of new models from the GEs of anomaly data is similar as explained in Section 5.2.1. Once the model detects the global anomaly at the model’s discrete abstraction level, the GEs produced from the local anomaly signal will be clustered. This information will be used first to update the individual transition probability matrices. Then the changes will be updated to the next level of joint vocabulary (words) and joint transition probability matrix (represented by green arrows in Figure 2).

The example of the joint transition probability matrix learned during the initial model $M_{0}$ is shown in Figure 15. Figure 20 and Figure 25 compares the transition probability matrices between different models. If compared to the joint transition matrix estimated in the training phase (see Figure 15), the joint transition probability matrix estimated in the incremental learning of the new model during the test phase has a higher number of discrete level vocabulary variables and consequently more transitions between discrete variables. The difference in transition matrices of the training phase and each test phase shows anomalies. From each abnormal situation, new models will be learned from the anomaly data to represent that situation. Therefore, if the system encounters similar situations in the future, the model’s discrete abstraction level would be able to represent well the collective situation of the ego-things.

We have considered a small network of two ego-things to evaluate the proposed methodology in this work. However, the different datasets collected from collaborative tasks have been used to explain the interpretability feature by comparing intermediate results produced by the incrementally learned models. Different scenario datasets are able to test the model’s fitness under different circumstances. The model complexity is mainly related to the complexity of events that generate anomalies. If the difference in the graphs and transitions probability matrices between different models is large, computational complexity increases, and more terms are needed to describe the anomaly. In this work, the main focus is not the number of agents/ego-things; instead, we have focused on interpreting the anomaly generated by an interaction between two vehicles. However, the complexity grows exponentially if the number of nodes (agents) increases and all possible interactions are to be modeled. However, these aspects go beyond the scope of this paper.

A more extensive network that involves more nodes can be considered in the future. Furthermore, future work needs to consider efficient communication schemes to distribute sensory data/parameters in the network for the self-awareness and collective-awareness functionalities.

## 6. Conclusions and Future Work

This paper presented a method to develop an interpretable machine learning model for the agents’ network. The interpretable data-driven model exposes self-awareness and collective awareness functionalities to detect local and global anomalies. To make inferences at different model abstraction levels, we have used a D-MJPF. In this work, interpretability is closely related to the continual incremental learning of new models whenever the existing model detects an abnormal situation. The intermediate results related to interpretability are presented and discussed. The interpretability part is vital in describing how the models make abnormality detection decisions and how the anomaly data has been exploited to learn new models incrementally. The model has been tested with different scenario datasets and we performed a comparative analysis of the results obtained at models’ different abstraction levels.

Developing a framework that allows formalizing the interpretability was challenging. Moreover, it was hard to design successive experiments and record all the sensory data to test and compare different models. The proposed approach can be applied in self-aware agents, so the agents are provided with incremental learning capabilities. This paper provides a first-level idea about performing interpretability included in a model forming inside an agent. Another issue was to design experiments that demonstrated the feasibility and logic of the approach. The framework we developed was more straightforward in interpreting events/abnormalities than humans do. The considered dataset, such as rotor velocity, was tough to analyze. However, it was evident from the designed experiments that the rotor data changes occurred because of an emergency operation performed by the ego-things. Furthermore, this was able to be explained by the free energy models we have learned during the abnormal situations.

Future research directions could incorporate multi-sensory data to develop multi-modal interpretable ML models. Another critical research direction could be considering different communication protocols to exchange data/parameters among ego-things and compare the performances by giving importance to energy efficiency. Moreover, as future work, automatic classification of abnormalities can be considered to improve the model performances.

## Figures and Tables

**Table 1:** Activated node pairs (labels) and values.

Activated Node Pairs		Label	Label Values (4D)
iCab1	iCab2		
*a*	*e*	α	[5.353 −0.007 5.327 −0.003]
*c*	*e*	β	[4.642 0.001 5.327 −0.003]
*c*	*f*	γ	[4.642 0.001 4.596 −0.009]
*b*	*f*	δ	[3.925 −0.005 4.596 −0.009]
*b*	*d*	ϵ	[3.925 −0.005 3.801 −0.006]
*c*	*d*	ζ	[4.642 0.001 3.801 −0.006]

**Table 2:** Activated node pairs (labels) and values.

Sl No.	Matching of Vocabulary (Node Pairs)		Label
	Train: Task 1	Test: Task 2	
1	-	*a,f*	η
2	-	*a,g*	θ
3	*b,d*	*c,g*	ϵ
4	*c,d*	*b,g*	ζ
5	*c,f*	*b,f*	γ
6	*b,f*	*c,f*	δ
7	-	*a,d*	λ
8	*c,e*	*c,e*	β
9	-	*b,e*	ψ
10	*a,e*	*a,e*	α
